# Collection and Reporting of Adult Sexual Orientation and Gender Identity Data in Highly Cited Cardiovascular Research

**DOI:** 10.1161/CIRCOUTCOMES.125.012724

**Published:** 2026-01-09

**Authors:** George S. Ponodath, Hari Dewan, Trisha Saha, Sneha Sarah Thomas, Justin C. Yang, Dean J. Connolly

**Affiliations:** 1 UCL Medical School, Faculty of Medical Sciences, University College London, United Kingdom (G.S.P., H.D., T.S.), University College London, United Kingdom.; 2 Institute of Cardiovascular Science, Faculty of Population Health Sciences, University College London, United Kingdom (G.S.P.), University College London, United Kingdom.; 3 Division of Psychiatry, Department of Epidemiology and Applied Clinical Research (J.C.Y.), University College London, United Kingdom.; 4 Faculty of Medical Sciences, University of Southampton, United Kingdom (S.S.T.).; 5 Department of Health Services Research and Policy, Faculty of Public Health and Policy, London School of Hygiene and Tropical Medicine, United Kingdom (D.J.C.).; 6 National Addiction Centre, Institute of Psychiatry, Psychology and Neuroscience, King’s College London, United Kingdom (D.J.C.).

**Keywords:** bibliometrics, data collection, gender identity, health equity, policy

Lesbian, gay, bisexual, transgender, queer/questioning, and other people minoritized by sex, sexual orientation, or gender (LGBTQ+) people experience wide inequalities in health outcomes and health care access, largely driven by anticipated or enacted structural and interpersonal discrimination.^1^ These disparities are perpetuated when researchers fail to collect information on LGBTQ+ persons in a culturally competent manner or report sexual orientation and gender (gender identity and trans status) in a way that is inclusive.^2^ To enhance understanding and help address inequalities in cardiovascular outcomes, researchers would need to routinely report these characteristics in accordance with best-practice guidance.^3^ Highly cited research is often well-funded and impactful in shaping policy and practice, and the inclusion of more granular descriptions of LGBTQ+ people in these studies can be a matter of health equity. This study aimed to assess the frequency and quality of sexual orientation and gender data collection and reporting practices in highly cited cardiovascular health research. A protocol was preregistered on the Open Science Framework: https://osf.io/mtvdx.

The data that support the findings of this study (including reasons for exclusion of full texts and the data extraction form) are available from the corresponding author on reasonable request. Ethical approval was not required for this project as it used only publicly available data and involved no human participants or animals. This was a retrospective cross-sectional bibliometric study, adherent to the STROBE guidelines for cross-sectional research. The PubMed database was used to identify cardiovascular research because it is an authoritative index of medicine-related literature and has a robust application programming interface enabling large-scale bibliometric analyses. Pilot searches identified which thematic (“Heart Diseases” [MeSH ID: 68006331] OR “Vascular Diseases” [MeSH ID: 68014652]) NOT (“Cardiovascular Abnormalities”[MeSH: 68018376]) and methodological (“Epidemiological Studies”[MeSH: 68016021] OR “Observational Study” [MeSH ID: 68064888] OR “Clinical Trial” [MeSH ID: 68016430] OR “Comparative Study” [MeSH ID: 68003160] OR “Multicenter Study” [MeSH ID: 68016448] OR “Twin Study” [MeSH ID: 68018486] OR “Validation Study” [MeSH ID: 68023361]) MeSH terms, combined with the boolean operator “AND,” would adequately capture all relevant PubMed-indexed literature. Final searches were conducted on January 8, 2024. Only English language studies reporting original cardiovascular health research with at least 10 adult (≥18 years) human participants were included in the analysis. Both observational (eg, surveys) and experimental studies (eg, clinical trials) were eligible, and there was no restriction by study location. The 400 most highly cited records, defined as the records with the highest citation count within a calendar year, were identified using the Crossref registry. The validity of this approach was confirmed by comparing a random sample of 100 citation counts to the Web of Science tag for highly cited records (sensitivity 100%, specificity 78%). Each article was reviewed in full by 1 author, and reasons for exclusion were documented. A piloted, prespecified data abstraction table was populated with data from each included record, including whether sexual orientation and gender data were collected, and the methodology used (see protocol).

The search returned 143 907 records (126 297 unique). After 2000 highly cited records were selected, 263 were found ineligible on full text review, yielding a final sample of 1737 records, including 745 clinical trials. Two records (0.1%) measured and reported gender identity (Table). The remaining 1735 records either measured sex only, conflated gender and sex, or reported gender identity as a binary variable without providing documentation regarding its operationalization (ie, the question/prompt and response options given to participants). In these cases, gender identity was reported as not collected. Although this approach may have led to undercounting, it reflects the availability of LGBTQ+-inclusive data for future meta-analysis. No record reported participants’ sexual orientation.

**Table.**
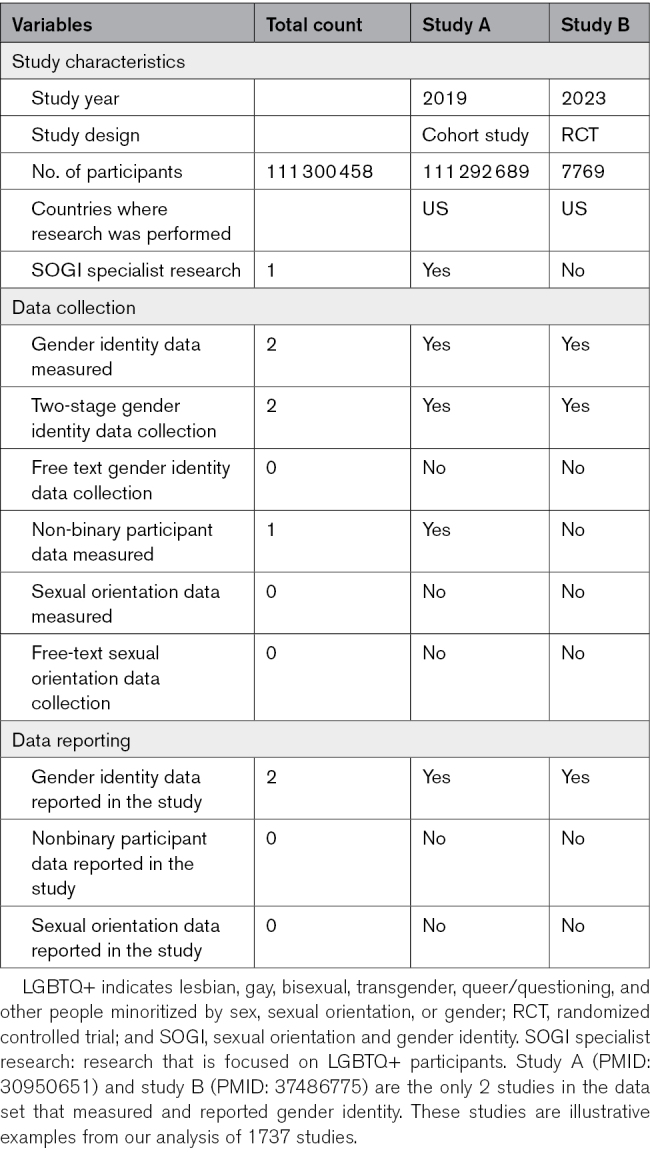
Characteristics of 2 Studies With Gender Identity Data Reporting and Counts of LGBTQ+ Inclusive Data Reporting Practices in the Data Set

To our knowledge, this is the largest analysis of sexual orientation and gender data reporting in cardiovascular research. Our findings indicate that detailed information on LGBTQ+ populations’ sexual orientation and gender identity remains unreported in highly cited cardiovascular health research. Only 1 randomized controlled trial collected gender identity, even though LGBTQ+ adults make up 9.3% of the population across North America.^4^ Although the collection of sexual orientation and gender data may not always be practical for a given study, the paucity of this data in highly cited cardiovascular research, especially in randomized controlled trials (which often dictate clinical practice), can contribute to disparities in cardiovascular health outcomes by omitting important patient information.^5^ This could lead to informational erasure of LGBTQ+ populations in research and reinforce structural discrimination^1^ and health care inequalities. Even in instances where LGBTQ+ subgroups are small, basic descriptive statistics could allow for future meta-analyses, including potential individual participant data meta-analysis. Our study has limitations. Due to resource constraints, data extraction was not performed in duplicate. Furthermore, we recognize there may be situations when patients are sensitive to the collection of this information if they see the study as unrelated. However, refusal rates for questions related to sex, sexual orientation, and gender are low, and community-informed, validated measures have been published.^3^

## ARTICLE INFORMATION

### Sources of Funding

None.

### Disclosures

None.

## Supplementary Material
